# Fabrication and Characterization of Silicon-Based Antimonene Thin Film via Electron Beam Evaporation

**DOI:** 10.3390/ma17051090

**Published:** 2024-02-27

**Authors:** Tingting Zhong, Lina Zeng, Junfeng Yang, Yichao Shu, Li Sun, Zaijin Li, Hao Chen, Guojun Liu, Zhongliang Qiao, Yi Qu, Dongxin Xu, Lianhe Li, Lin Li

**Affiliations:** 1 College of Physics and Electronic Engineering, Hainan Normal University, Haikou 571158, China; 20213085400009@hainnu.edu.cn (T.Z.); zenglina@hainnu.edu.cn (L.Z.); 20212070200004@hainnu.edu.cn (J.Y.); 20213085400001@hainnu.edu.cn (Y.S.); lisun_2014@163.com (L.S.); lizaijin@hainnu.edu.cn (Z.L.); 15948713468@163.com (H.C.); 068006@hainnu.edu.cn (G.L.); qzhl060910@hainnu.edu.cn (Z.Q.); quyi@hainnu.edu.cn (Y.Q.); 2 Key Laboratory of Laser Technology and Optoelectronic Functional Materials of Hainan Province, Hainan Normal University, Haikou 571158, China; jilinchangchun@yeah.net; 3 Hainan International Joint Research Center for Semiconductor Lasers, Hainan Normal University, Haikou 571158, China; lilianhehnnu@126.com

**Keywords:** antimonene, electron beam, deposition rate, Ge/Si substrate

## Abstract

Antimonene has attracted much attention due to its excellent characteristics of high carrier mobility, thermoelectric properties and high stability. It has great application prospects in Q-switched lasers, laser protection and spintronics. At present, the epitaxy growth of antimonene mainly depends on molecular beam epitaxy. We have successfully prepared antimonene films on silicon, germanium/silicon substrates for the first time using electron beam evaporation coating and studied the effects of the deposition rate and substrate on the preparation of antimonene; film characterization was performed via confocal microprobe Raman spectroscopy, via X-ray diffraction and using a scanning electron microscope. Raman spectroscopy showed that different deposition rates can lead to the formation of different structures of antimonene, such as α phase and β phase. At the same time, it was found that the growth of antimonene is also affected by different substrates and ion beams.

## 1. Introduction

Since graphene was successfully stripped [1], the two-dimensional (2D) materials of graphene have attracted people’s attention. The excellent gate control ability of 2D material makes it a promising channel material [2]. The thickness is usually smaller than that of the average free path of most particle transports, which forces two-dimensional materials to follow ballistic transport, and this quantum confinement effect fundamentally alters the electronic properties of two-dimensional thin film materials and is ideal for basic research and new electronic applications. In 2015, Zhang et al., for the first time, reported the theoretical results of two-dimensional antimonene of the five main group elements, of which their monolayer antimonene has a large band gap of 2.28 eV [3]. The high carrier mobility [4,5,6], stability [7], excellent thermoelectric performance, large band gap and spin-orbit coupling of antimonene have also been gradually verified, and it has potential in electron, photoelectron and spintronic research [8,9]. Using first principle calculation and analysis, antimonene can be tuned to a two-dimensional topological insulator (TI) under in-plane anisotropic strain [10]. The electronic properties of antimonene are regulated by uniaxial stretching [11]. The tunable bulk bandgap makes antimonene a promising material for achieving the quantum spin Hall effect (QSH) at high temperatures, meeting the requirements of future low-power electronic devices [12]. The β-Sb bilayer can be transformed from semi-metal to superconductor through electron doping [13]. The antimonene/graphene composite sponge is used as a pressure sensor, which has been successfully applied in flexible wearable sensors and human motion monitors with good sensitivity, high flexibility and a high mechanical, temperature and sensing stability [14]. In addition, antimonene is a promising energy storage material [15,16]. At the same time, antimonene can be used in surface plasmon resonance (SPR) sensors [17]. Raikwar et al. [18] used the transfer matrix method (TMM) to numerically analyze the sensor. The results show that the sensitivity of antimonene as a biomolecular recognition element layer (BRE) is 5% higher than that of traditional SPR biosensors. The sensitivity of a hybrid structure of MXene and antimonene can be improved to as high as 178.64°/RIU (refractive index unit). Similarly, with a binding layer combined with titanium dioxide, the sensitivity can reach 224.26°/RIU, which is 33.7% higher than that of a conventional SPR sensor. Xue et al. [19] assembled antimonene nanosheets on the surface of a gold chip using layer-by-layer technology and found that the sensitivity of antimonene to the SPR sensor varies with different thicknesses, which is defined as the ratio of the change in resonance angle to the change in the refractive index of the analyte, which is at least 2.3 times higher than existing miRNA sensors, even many orders of magnitude higher. It has a high refractive index in ultraviolet [20] and nonlinear characteristic behaviors [21,22] in optical properties and has applications in lasers [23,24] and laser protection [25].

**Figure 1:**
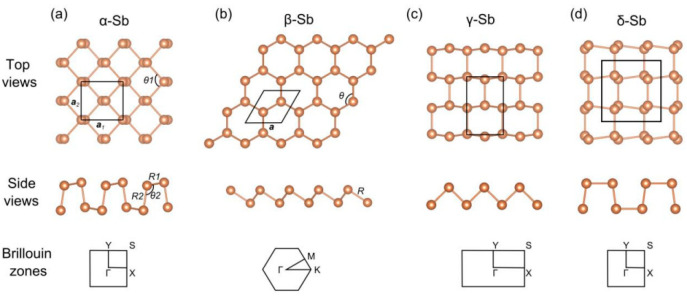
Multiple structures of antimonene: (**a**) α phase; (**b**) β phase; (**c**) γ phase; (**d**) δ phase [26].

Monolayer antimonene has many structures, including α, β, γ and δ phases, as shown in Figure 1. The α phase has two atomic layers, upper and lower, and the two atomic layers are not in the same plane. The β phase is the most stable structure, which has a hexagonal honeycomb structure similar to graphene. Wang et al. [26] studied the stability of antimonene by calculating the phonon dispersion curve. The antimony phonon dispersion curves of the α and β phases have no vibration mode, so the α and β phases are stable, while the γ and δ phases are not stable. Gupta et al. [27] also calculated the thermal conductivity of the α and β phases, and at the same temperature and edge roughness, the α phase has better thermal conductivity. The synthesis methods of antimonene include mechanical exfoliation [28], liquid exfoliation [29,30], epitaxial growth [31,32], etc. The mechanical exfoliation process is simple, and the cost is low, but the adhesive tape will take away a large number of flakes, resulting in a low stripping efficiency, and the stripping level is not easy to control, and the repeatability is poor. The liquid phase exfoliation method is a simple process not only with low cost, but also with a relatively large film size. However, this method is greatly affected by the solvent, ultrasound and other factors. Due to the influence of residual solvent, capillary and adhesive force, the thickness of the prepared antimonene is high during detection. The molecular beam epitaxy (MBE) method has good repeatability, high crystallinity, and in situ monitoring capabilities, but MBE equipment is expensive with relatively low yield [33]. Kuriakose et al. prepared monocrystals of antimonene on SiO_2_/Si substrate via van der Waals epitaxy and studied that antimonene exhibited a wide band transmittance in the visible to infrared range and light absorption peaks in the ultraviolet range [34]. In this study, antimonene thin films were prepared via electron beam evaporation. The variables affecting the growth of the antimonene thin films were easy to control, and the yield was large. The prepared antimonene thin films had good crystallization and high uniformity.

## 2. Experiment, Results and Discussion

### 2.1. Selection of Substrate

The lattice constants of the substrate and the epitaxial film need to match each other to a certain degree. They are completely coherent when the lattice mismatch number |δ| < 5%, have a semi-coherent interface when 5% < |δ| < 25% and completely mismatched when |δ| > 25%. After analysis, Si (the lattice mismatch of 8.4%) was selected as the substrate, which has a lower cost, has a mature process, and is commonly used in the industry. At the same time, Ge with an only 0.6% mismatch with the antimony lattice was selected as the transition layer to reduce the mismatch between Si and Sb.

The conditions of a high vacuum of 10^−4^ Pa, cavity temperature of 200 °C and deposition rate of 0.2 Å/s were set to prepare germanium thin films. The Ge atoms formed a buffer layer on the surface of the Si substrate via electron beam evaporation and formed a film via free diffusion. In the subsequent experiments, we used it as Ge/Si substrate for the preparation of antimonene.

### 2.2. Experimental Preparation and Result Discussion of Antimonen

**Figure 2:**
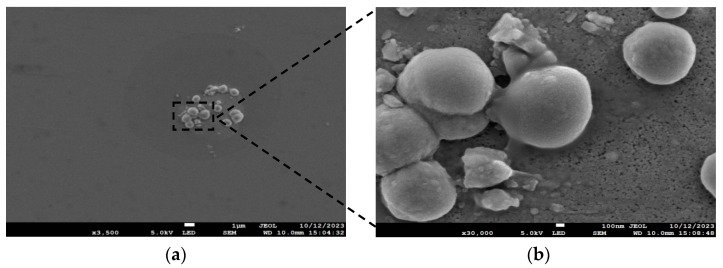
(**a**) SEM image of Ge/Si substrate at a deposition rate of 100 Å/s; (**b**) Enlarged picture, 8.57 times in the box.

The instrument used in this experiment was the ion source-assisted electron beam evaporation coating machine (Xinnan Technology Chengdu, Chengdu, China, ZZS720), and the discharge current (ion beam current) of the ion source was adjusted from 0 to 5 A. The antimony particles were volatile, and the deposition rate was 100 Å/s when the electron gun power was 6.8 W; the cavity temperature was 100 °C, and the ion beam current was 3 A. At this deposition rate, antimonene film is not easy to form, and antimony particles are easily attached to the substrate. The scanning electron microscope (SEM) used in the experiment was JEOL Ltd, Tokyo, Japan, JSM-7100F. As shown in Figure 2, using the scanning electron microscope, it was observed antimony particles completely covered the substrate surface with clusters of antimony attached above them. If the deposition rate is too large, the rate of particles colliding with each other is also large, resulting in a poor timely expansion ability of the particles, which are more likely to accumulate to form islands and grow like islands, that is, three-dimensional growth at this rate.

**Figure 3:**
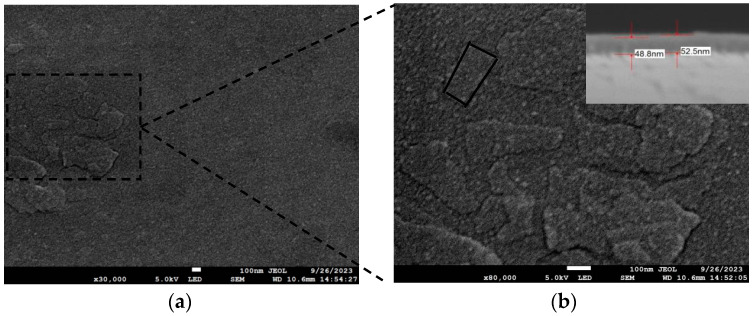
Under the experimental conditions of group A, the deposition rate on the Si substrate was 6.0 Å/s—SEM scanning surface and cross-section diagram: (**a**) SEM scan, enlarged image 30,000 times; (**b**) the same image enlarged 2.67 times in the box, the upper right is its cross-section.

The deposition rate was controlled through the electron gun power and spot size. Under a high vacuum of 10^−4^ pa, when the power of the electron gun was adjusted to 1.8 W, the cavity temperature was 100 °C, and the ion beam current was 3 A; the experimental conditions are shown in Table 1. The deposition rate of antimony on the Si substrate was 6.0 Å/s. As shown in Figure 3, the surface of the antimonene film produced on the Si substrate has triangular, rhomboid, trapezoidal and hexagonal structures, and its morphology has an obvious fractal structure and the appearance of steps. It can be seen from SEM cross-section scanning that the uniformity is good, and the thickness of the prepared film is about 50.0 nm. Most of the products prepared by Ji et al. [4] on a Si substrate via van der Waals epitaxy were particulate antimony. In our case, however, we used electron beam evaporation on a Si substrate, and what we obtained was antimonene with various edge structures, which is consistent with Ji et al.’s products on mica substrate, further indicating that 2D antimonene can be grown on Si substrate via electron beam evaporation. The force between mica and antimony is van der Waals force, and the antimony atomic clusters on the mica surface are not bound by the unbonded atoms on the substrate surface, and can achieve free migration, that is, mica can be used as the growth base of antimony, but the practical application of mica substrate is limited.

**Figure 4:**
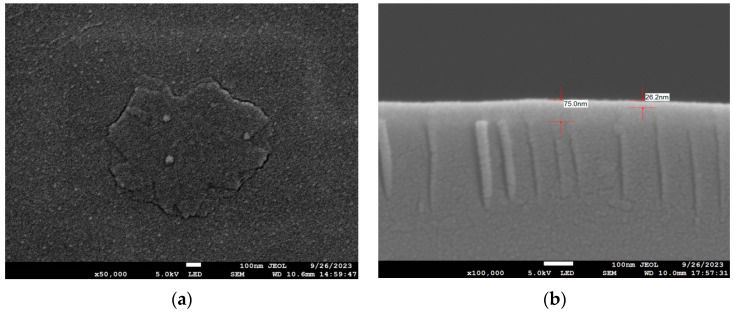
Under the experimental conditions of group B, the deposition rate on the Ge/Si substrate was 3.1 Å/s—SEM scanning surface and cross-section diagram: (**a**) surface drawings; (**b**) sectional drawings.

Under the same set of experimental conditions, the deposition rate on the Ge/Si substrate was 3.1 Å/s, and its morphology also had an obvious fractal structure, as shown in Figure 4a, whose fluctuation is lower than that of the Si substrate. The main stem grew from the central point, and the main stem formed a fractal structure with self-similar characteristics, and finally, the antimonene film was formed through aggregation and connection. Through scanning electron microscope images, it can be found that all fractal structures have clusters or a high thickness at their central points, which can be explained by the theory of Diffusion-Limited Aggregation [35]. As shown in Figure 4b, from the SEM cross-section, the 26.2 nm antimonene film was grown on the Ge/Si substrate, and its uniformity was good. It can be seen that the deposition rate and film thickness on the Ge/Si substrate are lower than those on the Si substrate under the same experimental conditions. Fortin-Deschênes et al. [7] used molecular beam epitaxial to prepare antimonene thin films on a Ge substrate. When the deposition temperature was less than 140 °C, the LEED image shows that the coating layer on the Ge substrate showed an amorphous structure; when the temperature was higher than 200 °C, the crystal structure corresponding to 2D antimony was generated on the Ge substrate surface, and the antimony crystal structure with a more regular shape was grown at high temperatures such as 325 °C. However, a high temperature will affect the adhesion of the Sb_4_ molecules. The structures of antimony crystals produced via electron beam evaporation under lower temperatures are similar to those at 325 °C, both show snowflake structures.

**Figure 5:**
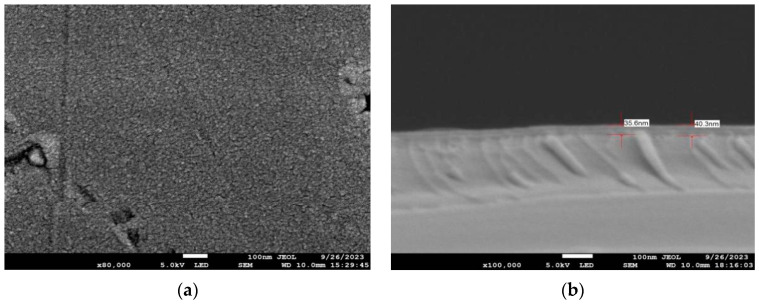
For the group A1 experiment, the SEM scanning surface and cross-section of Si substrate with a deposition rate of 6 Å/s and annealed for 30 min at 200 °C: (**a**) surface; (**b**) cross-section.

In order to study the effect of annealing on the growth of antimonene, samples of groups A and B were annealed at 200 °C, 300 °C and 400 °C for 1800 s. The experimental conditions are shown in Table 2. As shown in Figure 5, after annealing at 200 °C on the Si substrate, the surface of the film became more uniform and had a lower fluctuation than that of the unannealed one. The film behaved as a continuous film, and the annealed atoms migrated again, with a thickness of 35.6 nm~40.3 nm. In this case, however, there were also some defects, such as indentation.

**Figure 6:**
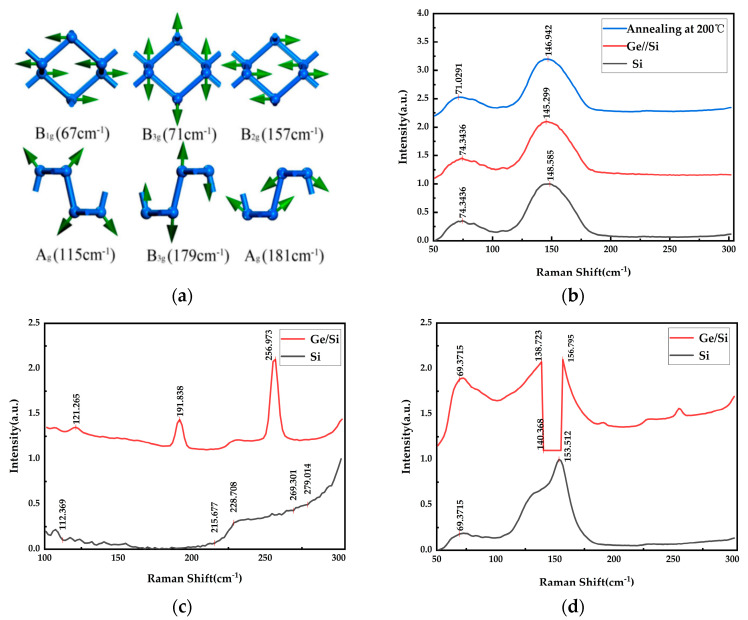
(**a**) Approximate Raman spectral data of α-phase antimonene simulated by means of first principle calculations; (**b**) Raman data for the group A, B and A1 experiments; (**c**) Raman diagram of the A2 and B2 experiments; (**d**) Raman diagrams for groups A3 and B3.

**Figure 7:**
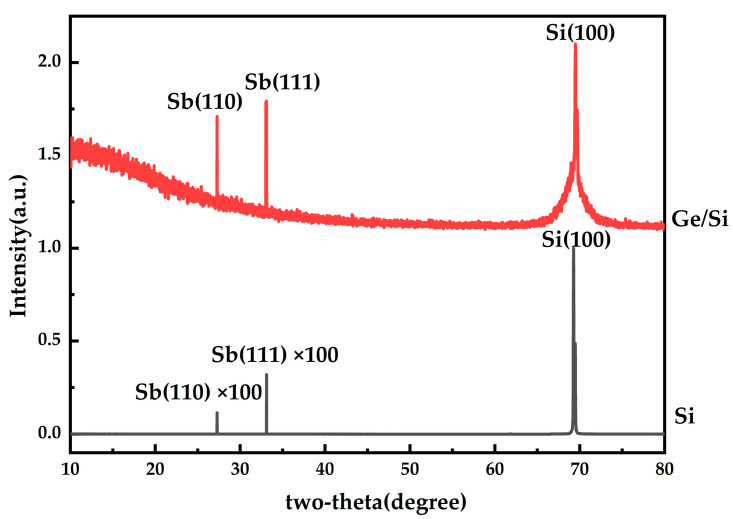
XRD image of Si substrate and Ge/Si substrate with deposition rates of 6.0 Å/s and 3.1 Å/s in the group A and B experiments.

The approximate Raman spectral data of α-phase antimonene simulated according to the first principle calculation [4] are shown in Figure 6a. α-antimonene has six vibration peaks: at 67 cm^−1^, 71 cm^−1^, 115 cm^−1^, 157 cm^−1^, 179 cm^−1^ and 181 cm^−1^. The Raman spectrometer used in the experiment was RENISHAW, British, London, InViaQontor. This is obviously corresponding to the vibration peaks of the 71 cm^−1^ and 145–148 cm^−1^ epitaxial antimonene obtained through experimental tests; as shown in Figure 6b, it can be determined that the epitaxial antimonene prepared under this experimental condition has a α-phase structure. The Raman peak value of the experimental group annealed at 200 °C showed a redshift compared with that of the unannealed group. This phenomenon may be caused by the change of film thickness after annealing [21], while the Raman data of the experimental group changed greatly at 300 °C and 400 °C. As shown in Figure 6d, the Raman peak of antimony cannot be found after annealing at 400 °C, and its peak value is relatively chaotic. In the case of the Ge/Si substrate, the Raman peak of Ge was 256.973 cm^−1^. It can be seen that at the annealing temperature, antimonene was easily volatilized, exposing the transition layer germanium, which was not conducive to the growth of antimonene, that is, high-temperature annealing is not conducive to the growth of antimonene. The X-ray diffraction (XRD) used in the experiment was Rigaku, Tokyo, Japan. X-ray diffraction, as shown in Figure 7, successfully detected Sb(110) and Sb(111) crystal faces at around 27.28° and 34.02°, respectively. In other words, the crystal structure of antimonene was successfully prepared under this experimental condition. The content of the antimony crystal structure of the Ge/Si substrate was higher than that of theh Si substrate, indicating that the Ge/Si substrate is more conducive to the growth of antimonene, and antimonene is dominant, while for the Si substrate, a lower content of antimony crystal could be obtained, and antimony was dominant.

**Figure 8:**
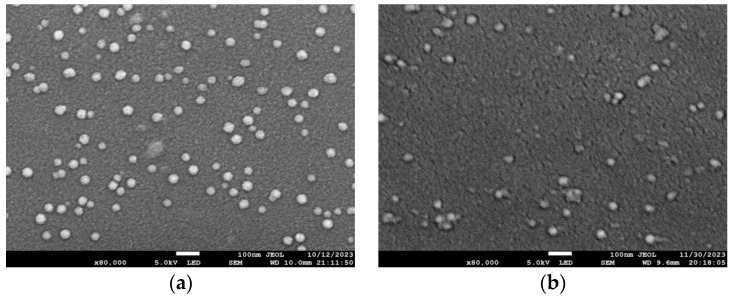
The deposition rates of antimony atoms on the Si substrate and Ge/Si substrate were 0.7 Å/s and 0.5 Å/s, respectively, and the thicknesses were about 10 nm: (**a**) group C experiment; (**b**) group D experiment.

Under the conditions of constant electron gun power, cavity temperature and ion beam current, the deposition rates on the Si substrate and Ge/Si substrate were reduced to the low rates of 0.7 Å/s and 0.5 Å/s, respectively, by enlarging the spot, experimental conditions are shown in Table 3. As shown in Figure 8, the thickness of the prepared film was about 10 nm. It was mainly in the form of clusters, a large number of islands in the form of tridimensional growth, attached to the substrate above, and the clusters on the Ge/Si substrate were less than on Si substrate. That is, antimonene with a low film thickness was not easy to generate.

**Figure 9:**
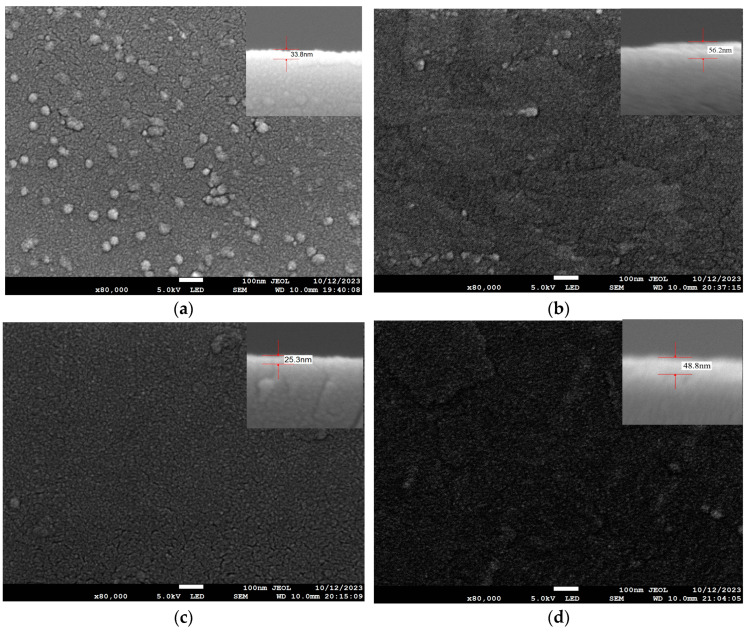
The deposition rates of the Si substrate and Ge/Si substrate were 0.7 Å/s and 0.5 Å/s, and the SEM scanning image, the top right, is the cross-section: (**a**) group E experiment, SEM image with a coating thickness of 20 nm set on the Si substrate; (**b**) group G experiment, SEM image with a coating thickness of 50 nm set on the Si substrate; (**c**) group F experiment, SEM image with a coating thickness of 20 nm set on the Ge/Si substrate; (**d**) group H experiment, SEM image with a coating thickness of 50 nm set on the Ge/Si substrate.

The system was set up to prepare films with thicknesses of about 20 nm and 50 nm. As shown in Figure 9a, in the experimental group of 20 nm on the Si substrate, its actual thickness was 33.8 nm, and a fractal structure gradually appeared, which is the coexistence of antimony and antimonene films. For the group with a thickness of 50 nm, the growth of fractal structures is shown in Figure 9b, that is, mutual migration occurs between antimony islands, forming thin films with low fluctuation. For the Ge/Si substrate, as shown in Figure 9c,d, the fractal structure of the antimonene film was more obvious than that of the Si substrate, and the antimony clusters are significantly reduced. When the thickness was 50 nm, there were basically no clusters, because the lattice mismatch between the Ge/Si substrate and antimony was small, the infiltration was good, and the deposited antimony atoms were more inclined to bond with the substrate atoms. As the film began to grow, the deposited atoms lay flat along the substrate. For film growth, it grows from bottom to top in the layered growth mode. It can be seen that it is easier to grow films on a Ge/Si substrate in two-dimensional growth mode than on a Si substrate under the same experimental conditions.

**Figure 10:**
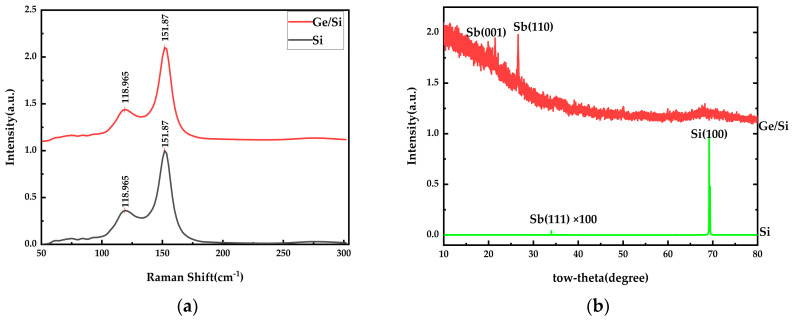
Group E and F experiments: Raman and XRD images of the Si substrate and Ge/Si substrate with deposition rates of 0.7 Å/s and 0.5 Å/s, respectively, and a thickness of about 20 nm: (**a**) Raman images; (**b**) XRD images.

As shown in Figure 10a, the Raman peaks were 118.965 cm^−1^ and 151.187 cm^−1^. According to the studies above and Fortin-Deschênes et al. [7], these peaks correspond to Eg and A1g peaks, which are typical of β-phase antimonene. It can be seen that the structure of antimonene generated at this rate is not consistent with that of the antimonene generated at 3.1 Å/s and 6.0 Å/s, that is, the deposition rate has a great influence on the formation of the antimonene structure. As shown in Figure 10b, the Sb(111) peak on the Si substrate is relatively weak and has poor crystallinity. Combined with the SEM results, it is mainly adhered to the Si substrate with antimony particles. The antimony peak on Ge/Si substrate is relatively strong and is dominated by a Sb(110) crystal surface. In combination with the SEM images, it can be inferred that the content of the antimonene film on the Ge/Si substrate increases and the content of antimony particles decreases under this condition.

In summary, it is not easy to form thin antimonene films on a Si substrate at low deposition rates. For further study, the experimental conditions are shown in Table 4, such as adjusting the cavity temperature to 200 °C and changing the ion beam current.

**Figure 11:**
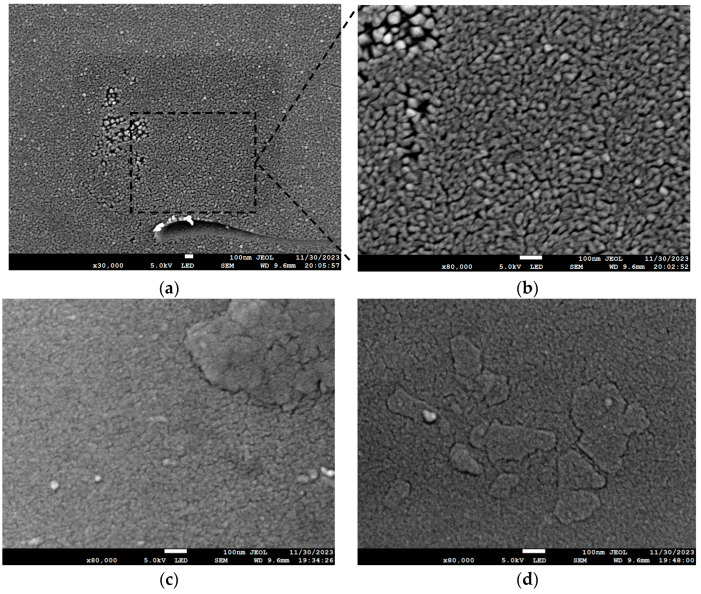
SEM image of Si substrate: (**a**) SEM diagram of experimental conditions in group I1; (**b**) the picture enlarged 2.67 times in the box; (**c**) SEM diagram of experimental conditions in group J1; (**d**) SEM diagram of experimental conditions in group K1.

For the group I1 experiment, a SEM result is shown in Figure 11a. When other conditions remain unchanged and the cavity temperature was 200 °C, the cluster islands mainly grew on the Si substrate, and the crystallinity was poor. In the group JI and K1 experiments, the effects of the ion beam on the growth of antimonene films at low deposition rates were studied. As shown in Figure 11c,d, when the ion beam current was 2 A, triangular and trapezoidal fractal structures appeared on the Si substrate, and antimonene films could be easily grown.

**Figure 12:**
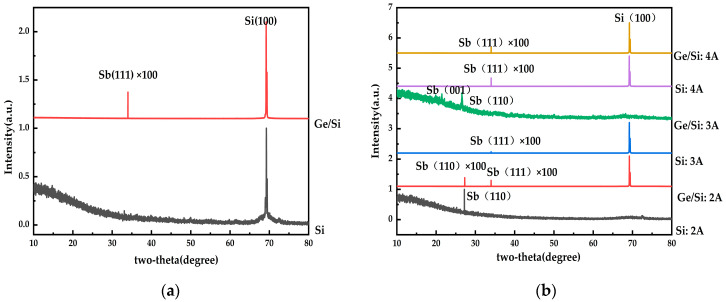
XRD patterns for a film thickness of about 20 nm: (**a**) the cavity temperature is 200 °C; (**b**) XRD images of different ion beams.

The XRD results of the group I1 experiment are shown in Figure 12a. The antimony signal on the Si substrate was not detected, that is, it was amorphous on the Si substrate. The peak value of the Sb(111) crystal surface on the Ge/Si substrate was very weak, that is, the crystallization of antimony on the Ge/Si substrate was very poor. It can be seen that when the cavity temperature was 200 °C, a mainly amorphous state appeared. That is, such a higher temperature is not conducive to the generation of antimonene. The XRD peaks under different ion beams are shown in Figure 12b. On the Si substrate, the ion beams were 3 A and 4 A, and existed in the Sb(111) crystal plane, and the peak intensity was low. However, there was a strong Sb(110) crystal surface when the ion beam was 2 A, which was a single crystal structure, indicating that the film had formed in the form of antimonene under an ion beam of 2 A, and its crystallinity was high. On the Ge/Si substrate, there was a strong antimony peak on the Sb(110) crystal surface when the ion beam current was 3 A. At ion beams of 2 A and 4 A, there were also antimony peaks, but their peak intensities were relatively weak, that is, the content of antimonene films on Ge/Si substrates is the highest at the ion beam of 3 A, while antimony is dominant at 2 A and 4 A.

**Figure 13:**
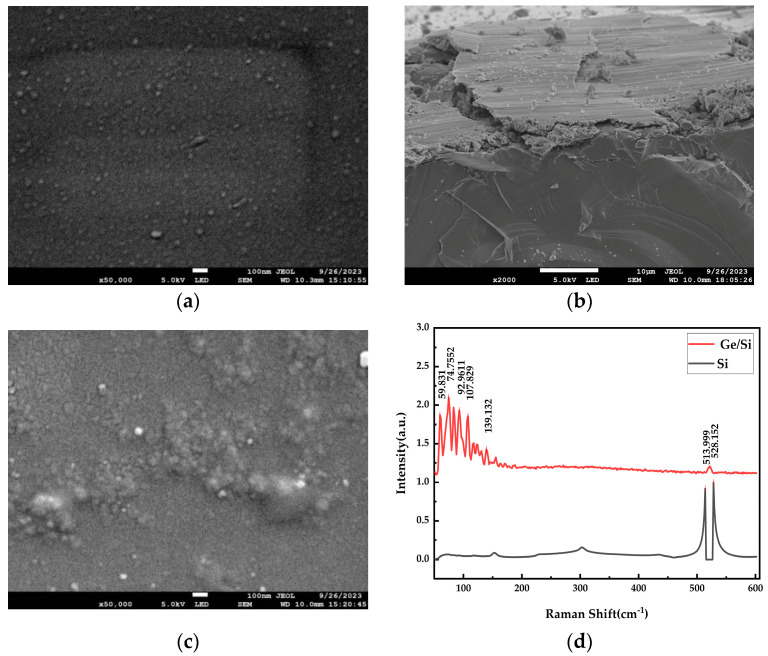
When the deposition rate is 0.1 Å/s, a SEM scan and Raman map were obtained: (**a**) group L experiment, SEM scanning surface diagram on the Si substrate; (**b**) group L experiment, cross-section of SEM scanning on the Si substrate; (**c**) group M experiment, SEM scanning surface diagram on the Ge/Si substrate; (**d**) Raman images of group L and M experiments.

When we changed the experimental conditions of the cavity temperature to 100 °C and the ion beam current to 3 A, the power of the electron gun was adjusted to 1.6 W, and the deposition rate was reduced to 0.1 Å/s. As shown in Figure 13, antimony was not found on the substrate surface, that is, at this power, antimony is not easy to volatilize, and the actual deposition rate of antimony is zero. At the same time, the Raman spectra was studied, as shown in Figure 13d. Only the Raman peak of silicon existed on the Si substrate, and there was no Raman peak of antimony, which further indicates that antimonene cannot be generated under this condition.

**Figure 14:**
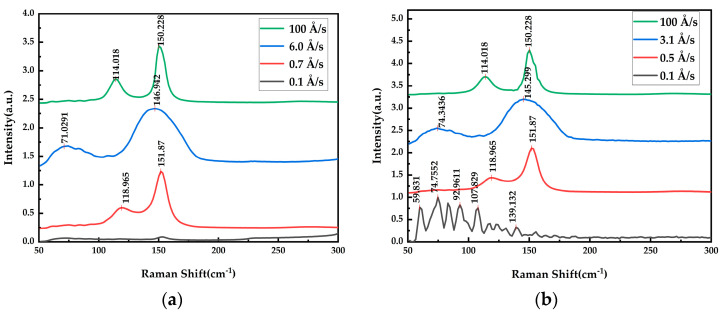
Raman images of films for thicknesses of about 20 nm at different deposition rates: (**a**) Si substrate; (**b**) Ge/Si substrate.

The antimonene produced using different deposition rates was further studied, as shown in Figure 14. It can be seen that the structure of the antimonene produced at different deposition rates was different. When the deposition rate of the Si substrate was 100 Å/s and 0.7 Å/s, the structures were β phase, and α phase at 6.0 Å/s. In the case of 0.7 Å/s, compared with 100 Å/s, the Raman peak was blue shifted; the reason may be that, in the case of 100 Å/s, three-dimensional growth dominates, and at the same time, the thickness of the growth is easier to control and thinner at the low deposition rate of 0.7 Å/s. The same characteristics also existed for different deposition rates on the Ge/Si substrates.

## 3. Conclusions

It was found that the preparation of antimonene thin films via electron beam evaporation is affected by the substrate, deposition rate, cavity temperature and ion beam current. Compared with a Si substrate, a Ge/Si substrate has a smaller lattice mismatch with antimony and better infiltration, which is more favorable for layer growth, that is, more conducive to two-dimensional antimonene growth. At lower deposition rates, when the ion beam current is 3 A, antimony particles are mainly attached to the Si substrate, and thin antimonene/films are not easy to form, and antimony mainly appears in the shapes of islands. When the ion beam current is 2 A, the antimonene film is formed on the Si substrate, which has a single crystal structure and good crystallinity. When the cavity temperature is 200 °C, the film has an amorphous structure, which is not conducive to the formation of antimonene. At low deposition rates, the growth of antimonene on a Si substrate is easier at a 100 °C cavity temperature and 2 A ion beam. The deposition rate also has a great influence on the growth of antimonene films, and different deposition rates lead to the growth of different phases of antimonene. In other words, the stacking forms of antimonene are different under different deposition rates. Therefore, antimonene films with different structures and thicknesses can be grown according to the actual application requirements.

## Figures and Tables

**Table 1:** Experimental conditions of group A and group B.

	Substrate	Deposition Rate (Å/s)	Time (s)	Thickness (nm)
A	Si	6.0	84	50
B	Ge/Si	3.1	84	26

**Table 2:** Experimental conditions for annealing.

	Substrate	Annealing Temperature (°C)	Annealing Time (s)
A1	Si	200	1800
A2	Si	300	1800
A3	Si	400	1800
B1	Ge/Si	200	1800
B2	Ge/Si	300	1800
B3	Ge/Si	400	1800

**Table 3:** Experimental conditions of different thicknesses and deposition rates.

	Substrate	Deposition Rate (Å/s)	Time (s)	Thickness (nm)
C	Si	0.7	228	16
D	Ge/Si	0.5	228	11
E	Si	0.7	400	28
F	Ge/Si	0.5	400	20
G	Si	0.7	815	57
H	Ge/Si	0.5	815	41

**Table 4:** Experimental conditions for the preparation of antimonene.

	Substrate	Electron Gun (W)	Cavity Temperature (°C)	Ion Beam Current (A)	Time (s)	Thickness (nm)
I1	Si	1.8	200	3	362	25
I2	Ge/Si	1.8	200	3	362	18
J1	Si	1.8	100	4	374	26
J2	Ge/Si	1.8	100	4	374	19
K1	Si	1.8	100	2	366	26
K2	Ge/Si	1.8	100	2	366	18
L	Si	1.6	100	3	2000	20
M	Ge/Si	1.6	100	3	2000	20

## Data Availability

Data are contained within the article.
